# Therapeutic effect of a TM4SF5-specific monoclonal antibody against colon cancer in a mouse model

**DOI:** 10.18632/oncotarget.2311

**Published:** 2014-08-05

**Authors:** Young-Eun Kim, Sanghoon Kwon, Guang Wu, Dongbum Kim, Byoung Kwon Park, Jeong-A Park, Kyung-Chan Choi, Doo-Sik Kim, Hyung-Joo Kwon, Younghee Lee

**Affiliations:** ^1^ Department of Biochemistry, College of Natural Sciences, Chungbuk National University, Chungbuk, Republic of Korea; ^2^ Center for Medical Science Research, College of Medicine, Hallym University, Gangwon-do, Republic of Korea; ^3^ Department of Microbiology, College of Medicine, Hallym University, Gangwon-do, Republic of Korea; ^4^ Department of Pathology, College of Medicine, Hallym University, Gangwon-do, Republic of Korea; ^5^ Department of Biochemistry, College of Life Science and Biotechnology, Yonsei University, Seoul, Republic of Korea

**Keywords:** TM4SF5, monoclonal antibody, colon cancer, therapeutics, animal model

## Abstract

Transmembrane 4 superfamily member 5 protein (TM4SF5) is presumed to serve as a molecular target to prevent or treat hepatocellular carcinoma (HCC) and colon cancer in a mouse model. Previously, we reported the efficacy of anti-cancer peptide vaccine targeting TM4SF5. In addition, we reported an anti-proliferative effect of anti-TM4SF5 monoclonal antibody in HCC. Here, we investigated expression of TM4SF5 in 45 primary colon cancer tissues. Almost all of the colon cancer tissues expressed TM4SF5 based on immunohistochemistry using anti-TM4SF5 monoclonal antibody. The treatment of human colon cancer cells with anti-TM4SF5 antibody reduced growth of TM4SF5 expressing cells and enhanced expression of E-cadherin and β-catenin. Using mouse colon cancer models, we then evaluated the *in vivo* anti-cancer effect of anti-TM4SF5 antibody. Injection of the antibody significantly reduced growth of tumors priorly established by subcutaneous injection of human colon cancer cells HT-29 in a xenograft setting. We obtained similar results with mouse colon cancer cell line CT-26 in an allograft setting. Therefore, we suggest that the TM4SF5-specific monoclonal antibody has a therapeutic effect against colon cancer.

## INTRODUCTION

Colon cancer is one of the most common cancers in the world. It has high mortality, about 30% ~ 40% in the United States and Europe, and is more common in developed countries than in undeveloped countries [1]. As the number of deaths from colon cancer is increasing worldwide, prevention, early diagnosis, and efficacious therapy are critically important [2]. Surgery, chemotherapy, and radiotherapy are generally performed, alone or in combination, to treat patients with colon cancer [3-5]. Immunotherapeutic approaches using peptide vaccines, dendritic cell-based cancer vaccines, whole tumor cell vaccines, viral vector-based cancer vaccines, adoptive cell transfer therapy, antibody-based cancer immunotherapy, and cytokine therapy are another treatment option [6]. Immunotherapy has gained attention as the therapeutics has narrow specificity and therefore may have fewer side effects [7-10].

Understanding the receptor-mediated signaling pathways involved in cancer development is important for designing cancer-specific therapeutics targeting extracellular molecules. Several well-known oncogenic pathways have been implicated in the pathogenesis of cancers, including the epidermal growth factor receptor (EGFR), hepatocyte growth factor receptor (HGF/c-Met), and vascular endothelial growth factor (VEGF) pathways [11-15]. Mutations in oncogenes and tumor suppressor genes involved in these receptor signaling pathways are commonly observed in several cancers including colon cancer [15,16].

In the past decade, small molecule inhibitors and antibodies targeting these receptors have been widely investigated in both preclinical and clinical applications to block the pathogenesis of cancers [17-21]. Bevacizumab (Avastin) is a recombinant humanized monoclonal antibody against VEGF that has proven to be effective for the treatment of colon cancer, renal cancer, ovarian cancer, etc. [19-21]. Other neutralizing monoclonal antibodies against EGFR (cetuximab and panitumumab) have been approved as therapeutics for colon cancer [22,23].

Tetraspanins, also known as transmembrane 4 superfamily (TM4SF) members, can associate with various molecules such as integrins, membrane receptors, immunoglobulin superfamily proteins, and other tetraspanins to form a multimolecular tetraspanin web [24,25]. Because of the great heterogeneity in the composition of the web, tetraspanins are widely involved in regulation of cell differentiation, activation, growth, and migration [26-30]. Recent reports revealed that tetraspanins function as both suppressors and promoters of metastasis depending on the particular tetraspanins [31]. Tetraspanins have thus gained attention as diagnostic and prognostic markers, as well as therapeutic targets for preventing tumor progression [31,32].

TM4SF5, one of the tetraspanins, was previously implicated in hepatocellular carcinoma (HCC) [33,34]. TM4SF5 is involved in epithelial-mesenchymal transition, loss of contact inhibition, and regulation of VEGF-mediated angiogenesis through cooperation with integrins [34,35]. TM4SF5 expression enhanced migration and invasion, which may contribute to effective metastasis [36]. Previously, we established a method to induce production of antibodies using peptide epitopes in combination with CpG-DNA-liposome complex without carriers [37]. Using this technology, we showed that TM4SF5 can serve as a molecular target for HCC and colon cancer: a peptide vaccine targeting TM4SF5 had preventive or therapeutic effects against HCC and colon cancer in mouse models [38-41]. Therefore, we postulated that TM4SF5-specific monoclonal antibody can serve as a therapeutic antibody to treat these cancers and we recently proved that injection of anti-TM4SF5 antibody can suppress the growth of HCC tumor in a mouse model [42].

In this study, we investigated expression of TM4SF5 in colon cancer tissues and the therapeutic effect of the TM4SF5-specific monoclonal antibody against colon cancer in a mouse model. We confirmed that injection of anti-TM4SF5 antibody reduced the growth of tumors derived from pre-implanted colon cancer cells in mice.

## RESULTS

### Expression of the TM4SF5 protein in human colon cancer tissues

**Figure 1:**
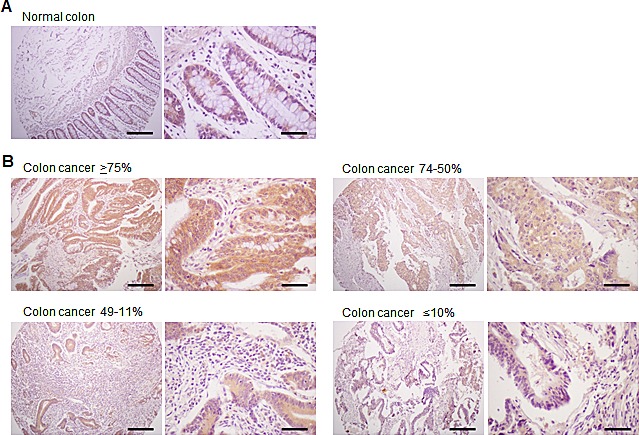
Expression of TM4SF5 in colon cancer tissues An immunohistochemical analysis of colon cancer tissues was performed with anti-TM4SF5 monoclonal antibody and colon cancer tissue arrays. A. Normal colon tissue. B. Examples of colon cancer tissues with ≥75%, 74-50%, 49-11%, and ≤10% of tumor cells expressing TM4SF5. Scale bars; left panel, 200 μm. right panel, 50 μm.

In order to investigate expression of the TM4SF5 protein in colon cancer tissues, we performed immunohistochemical staining with the anti-TM4SF5 monoclonal antibody. We analyzed 45 samples of primary colon cancer tissues along with normal colon tissue samples as a control (tissue microarray A203VII). As shown in Figure 1A, there was no expression of TM4SF5 in normal colon tissues. However, almost all of the colon cancer tissues (44 among 45 samples) expressed TM4SF5 (97.8%, staining in >11% of tumor cells) (Figure 1B, Table 1). Approximately 35.6% of colon cancer tissue samples expressed TM4SF5 in ≥75% of tumor cells and ~40% of colon cancer tissues were positive for TM4SF5 expression in 74-50% of tumor cells. It is thus concluded that expression of TM4SF5 can serve as a marker of colon cancer. However, the levels of TM4SF5 expression did not correlate with colon tumor grade or stage (Table 2).

**Table 1:** Immunohistochemical analysis of TM4SF5 expression in colon cancer tissues

Colon tissue sections (AccuMax Array)	n	TM4SF5 positive (%)	Number (%) of cases expressing TM4SF5			
			≥75%	74-50%	49-11%	≤10%
A203VII	45	97.8	16 (35.6)	18 (40.0)	10 (22.2)	1 (2.2)

Percentages in parentheses were calculated as the number of TM4SF5-psoitive samples for each quartile, divided by the total number of samples in each tumor type.

**Table 2:** Expression of TM4SF5 in colon cancer tissues A203 (VII) Colon cancer tissues

No.	Sex	Age	TNM stage	Grade	Ab staining
1	M	63	T3N0M0	G2	+++
2	M	33	T3N0M0	G2	++++
3	F	72	T3N0M0	G2	+++
4	M	72	T3N0M0	G2	++++
5	M	72	T3N1M0	G2	++
6	F	49	T3N0M0	G2	+++
7	F	67	T4N0M0	G2	+
8	M	51	T3N1M0	G2	+++
9	M	63	T2N0M0	G2	++
10	M	57	T3N0M0	G2	+++
11	M	65	T3N0M0	G2	++
12	F	48	T4N1M0	G2	++++
13	F	50	T3N2M0	G2	+++
14	M	27	T3N0M0	G2	++
15	F	39	T3N0M0	G2	++
16	F	69	T3N1M1	G2	++
17	F	51	T4N0M1		++++
18	M	50	T3N0M	G2	+++
19	F	77	T4N1M0	G2	++
20	F	77	T3N0M0	G2	++++
21	F	45	T3N2M0	G2	+++
22	M	58	T3N0M0	G2	++++
23	F	39	T3N0M0	G2	+++
24	F	47	T4N0M0	G2	+++
25	F	75	T3N0M0	G2	++++
26	F	71	T3N1M0	G2	+++
27	F	40	T4N2M1	G2	++
28	M	70	T4N1M1	G2	++++++
29	M	67	T3N0M0	G2	+++
30	M	43	T3N1M0	G2	++++
31	F	63	T4N0M0	mucinous	++++
32	F	53	T3N0M0	G1	+++
33	M	52	T3N1M0	G1	+++
34	M	59	T3N0M0	G2	++++
35	M	41	T4N2M0	G2	++++
36	M	56	T3N1M0	G1	++
37	M	64	T3N2M0	G1	+++
38	F	65	T1N0M0	G1	++++
39	M	69	T4N1M0	G2	+++
40	F	58	T3N1M0	G2	++++
41	M	57	T3N0M0	G3	++
42	M	56	T3N1M0	G3	+++
43	F	52	T3N2M0	G3	++++
44	F	53	T3N2M0	G2	++++
45	M	68	T3N0M0	G2	++++

≥75% (4, ++++), 50-74% (3, +++), 11-49% (2, ++), ≤10% (1, +)

### Expression of TM4SF5 in human colon cancer cell lines and the effect of anti-TM4SF5 antibody on the growth of TM4SF5 expressing cells

**Figure 2:**
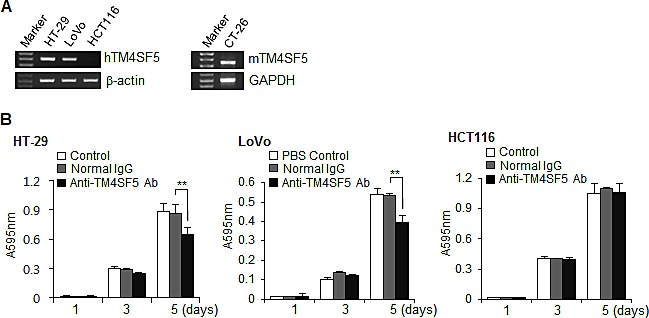
Expression of TM4SF5 in colon cancer cells and the effect of anti-TM4SF5 antibody on the growth of colon cancer cells A. The expression levels of TM4SF5 mRNA in the indicated human (left) and mouse (right) colon cancer cell lines were analyzed by RT-PCR. B. Effect of anti-TM4SF5 antibody on the growth of human colon cancer cells. Cell growth was measured by an MTT assay. Each bar is expressed as the mean + standard deviation of three experiments. **P< 0.01. (*vs* normal IgG control).

To validate TM4SF5 as an efficacious target for anti-colon cancer therapy, we first examined the expression of TM4SF5 mRNA in human colon cancer cell lines. As shown in Figure 2A, HT-29 and LoVo cells expressed human TM4SF5 mRNA, but HCT116 did not express TM4SF5. Therefore, these cell lines can be used as a model. Expression of TM4SF5 was also detected in a mouse colon cancer cell line CT-26, as previously reported [40], and therefore CT-26 cells were used for a mouse allograft model.

We then investigated the effect of the anti-TM4SF5 monoclonal antibody on colon cancer cell growth. The growth of HT-29 cells and LoVo cells expressing TM4SF5 was delayed by the antibody treatment. However, there was no change with control IgG treatment. In contrast, anti-TM4SF5 antibody did not induce a significant change in the growth of HCT116 cells, which did not express TM4SF5 (Figure 2B). Therefore, we conclude that the anti-TM4SF5 antibody has an anti-proliferative effect on human colon cancer cells expressing TM4SF5 *in vitro*.

### Effect of the anti-hTM4SF5 monoclonal antibody on the expression of E-cadherin and β-catenin in human colon cancer cells

**Figure 3:**
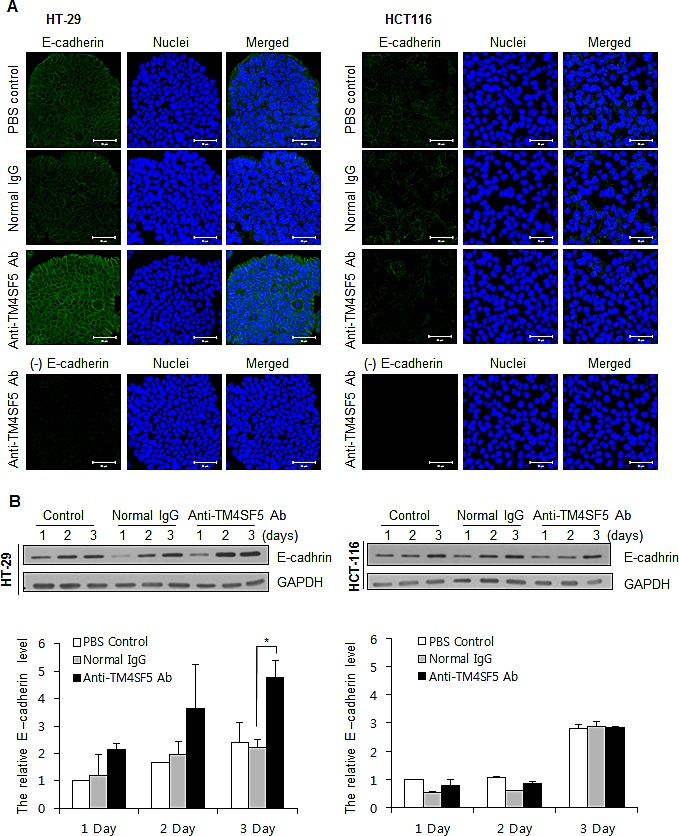
Enhanced expression of E-cadherin in TM4SF5 expressing cells after treatment with anti-TM4SF5 antibody A. The expression levels of E-cadherin in the colon cancer cells, HT-29 and HCT116 cells, were analyzed by confocal microscopy after treatment with PBS, normal IgG, or the anti-TM4SF5 monoclonal antibody for 3 days. The secondary antibody control without the anti-E-caherin antibody staining is shown as (-) E cadherin. B. (Top) The expression levels of E-cadherin in the colon cancer cells were analyzed by Western blotting. The expression levels of GAPDH are shown as a loading control. These are representative of at least three independent experiments. (Bottom) The band intensity was measured using Photoshop CS6 and normalized with the amount of GAPDH control. Relative expression levels of E-cadherin are shown taking the value of the PBS control group after treatment for 1day as 1.0. *p<0.05, mean ± SD.

E-cadherin is a major protein involved in cell-cell interaction. Previously, we found that treatment of HCC cells expressing TM4SF5 with anti-TM4SF5 antibody enhances expression of E-cadherin [42]. We therefore checked expression of E-cadherin using confocal microscopy and Western blotting analysis. As shown in Figures 3A and 3B, treatment of HT-29 cells with the anti-TM4SF5 antibody enhanced expression of E-cadherin compared to the untreated control or IgG-treated control. In contrast, there was no prominent difference in HCT116 cells.

**Figure 4:**
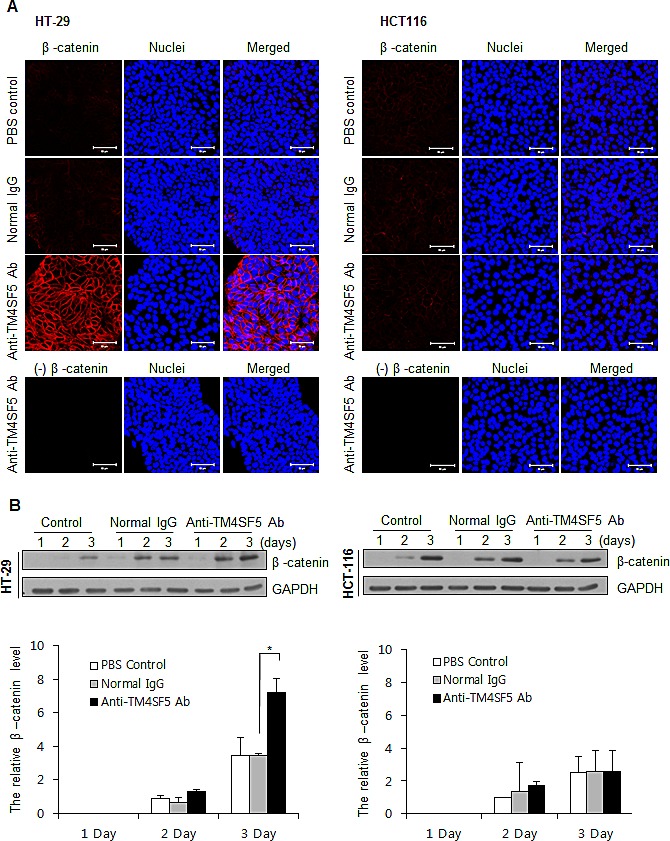
Enhanced expression of β-catenin in TM4SF5 expressing cells after treatment with anti-TM4SF5 antibody A. The expression levels of β-catenin in the colon cancer cells, HT-29 and HCT116 cells, were analyzed by confocal microscopy after treatment with PBS, normal IgG, or the anti-TM4SF5 monoclonal antibody for 5 days. B. (Top) The expression levels of β-catenin in the colon cancer cells were analyzed by Western blotting. The expression levels of GAPDH are shown as a loading control. These are representative of at least three experiments. (Bottom) The band intensity was measured and normalized with the amount of GAPDH. Relative expression levels of β-catenin are shown taking the value of the PBS control group after treatment for 2 days as 1.0. *p<0.05, mean ± SD.

In addition to E-cadherin, β-catenin is an essential component of the cell adhesion complex [43]. We therefore also investigated expression of β-catenin (Figure 4). Treatment of HT-29 cells with anti-TM4SF5 antibody clearly enhanced expression of β-catenin. However, there was no difference in the untreated control or IgG-treated control. Treatment of HCT116 cells with anti-TM4SF5 antibody induced no change. These results suggest that enhanced cell-cell interaction induced by treatment with TM4SF5 antibody contributes to contact inhibition in TM4SF5 expressing cells.

We previously showed that the anti-TM4SF5 antibody induced changes in actin polymerization and formation of focal adhesion sites in HCC cells based on phalloidin and paxillin staining [42]. However, we found that there was no change in colon cancer cells (data not shown). Other changes observed in TM4SF5 expressing HCC cells treated with anti-TM4SF5 antibody were nuclear translocation of the CDK inhibitor p27^kip1^ and increased activity of a small GTP protein RhoA [42]. Therefore, we checked these aspects and found that anti-TM4SF5 did not induce a prominent change in colon cancer cells (data not shown). Therefore, it is likely that the specific action mechanism of anti-TM4SF5 differs depending on the specific types of tissue expressing TM4SF5.

### Localization of the injected anti-TM4SF5 monoclonal antibody on colon tumors *in vivo*

**Figure 5:**
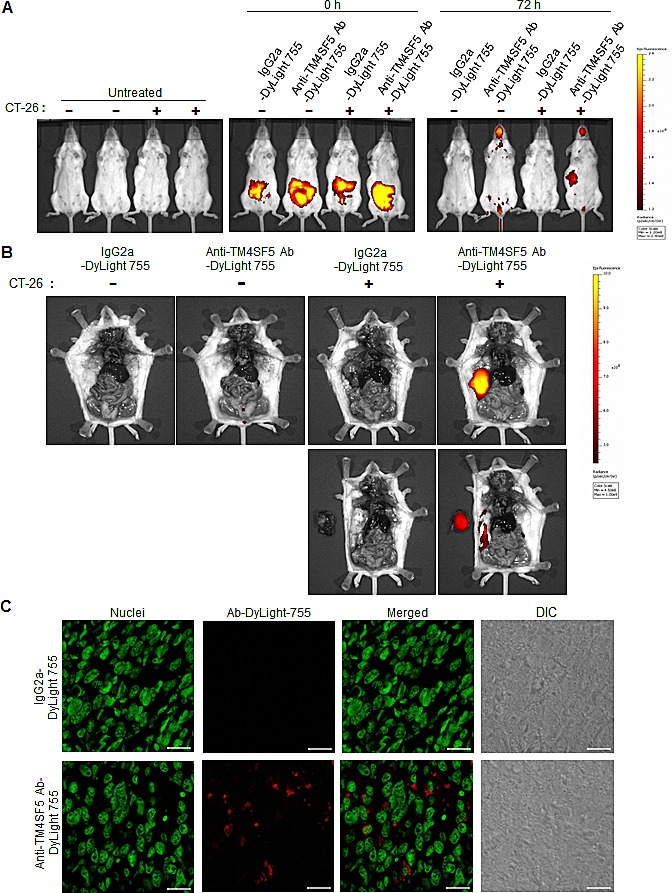
Biodistribution of the anti-TM4SF5 monoclonal antibody in colon tumor tissue BALB/c mice were injected with PBS as a control or CT-26 cells to generate tumor-bearing mice. A. DyLight 755-labeled IgG2a isotype control or the anti-TM4SF5 monoclonal antibody was injected into the intraperitoneal cavity of the mice, and the whole body fluorescence was examined using a real-time IVIS Imaging System 200 Series at the starting time point and after 72 h. B. The mice were dissected and the localization of the antibody was examined using the IVIS imaging system (upper panel). Tumor tissues were taken out and placed to the left of the mice (lower panel). C. The harvested tumor tissue was frozen, and the microsection samples were stained with SYTOX Green dye for nuclei staining and analyzed by confocal microscopy. Scale bars, 20 μm. These are representative of two independent experiments with four mice per each treatment.

As the anti-TM4SF5 monoclonal antibody can inhibit the growth of colon cancer cell lines *in vitro*, we next investigated the *in vivo* effect of the antibody on tumors. First, we determined the distribution of the anti-TM4SF5 antibody after injection into mice. The anti-TM4SF5 antibody and mouse IgG2a control were conjugated with DyLight 755 (a fluorescent dye) and the DyLight-labeled antibodies were injected into the intraperitoneal cavity of control mice or mice harboring CT-26 cell derived tumors. After 72 h, the distribution of the labeled antibody was quantified by measuring the total photon flux (photons/sec) of the fluorescence. As shown in Figures 5A and 5B, the DyLight 755-labeled anti-TM4SF5 monoclonal antibody was localized in the tumors, whereas the DyLight 755-labeled IgG2a control was not detected in the mice. When we cut out the tumor mass and analyzed microsections of the frozen tissue, we found that many of the tumor cells were stained with the DyLight 755-labeled anti-TM4SF5 antibody (Figure 5C). In contrast, we could not detect any labeling in the control sections obtained from mice injected with DyLight 755-labeled IgG2a control. Therefore, the anti-hTM4SF5 monoclonal antibody can target colon tumor cells expressing TM4SF5 *in vivo*.

### Anti-TM4SF5 monoclonal antibody inhibits growth of colon tumors in a xenograft mouse model

**Figure 6:**
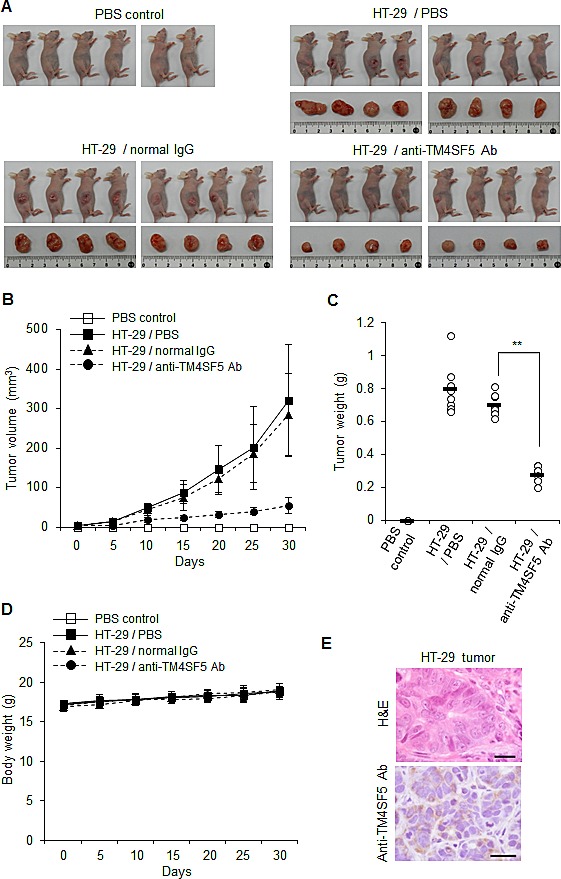
Therapeutic efficacy of the anti-TM4SF5 monoclonal antibody against colon tumor growth in a xenograft mouse model A mouse xenograft model was established by the implantation of HT-29 cells in BALB/cAnCrj-nu/nu mice. When the tumors reached 5 mm in diameter, PBS, normal IgG or the anti-TM4SF5 monoclonal antibody was injected into the mice and tumor growth was monitored for 30 days (n=8 each). Control mice injected with PBS are shown as a control (n=6). A. Macroscopic appearance of tumor bearing mice and dissected tumor tissues. B. Tumor volume (width^2^ x length/2). Data are represented as mean + standard deviation. C. Individual tumor weights for each treatment group. Mean values are indicated as a horizontal bar. D. Individual body weights for each treatment group. Data are represented as mean + standard deviation. E. Expression of TM4SF5 in the tumor tissues. TM4SF5 expression was confirmed by an immunohistochemical analysis using the anti-TM4SF5 monoclonal antibody. This image is representative of colon tumor tissues. The TM4SF5 positive area is expressed as a brown color. H&E and IHC represent hematoxylin and eosin staining and immunohistochemistry, respectively. Scale bars, 20 μm. **p<0.01, mean ± SD.

To evaluate the efficacy of the anti-TM4SF5 antibody against colon cancer in mice, we determined the effect of the anti-hTM4SF5 antibody on the growth of colon cancer cells *in vivo* using human cell line HT-29 and a xenograft mouse model. We injected nude mice subcutaneously in the dorsal right flank with HT-29 cells and allowed the tumors to grow. When the tumor size reached 5 mm in diameter, we injected the animals twice a week in the intraperitoneal cavity with PBS, normal mouse IgG, or anti-TM4SF5 monoclonal antibody. Based on the tumor volume and weight, anti-TM4SF5 monoclonal antibody attenuated the progression of colon tumors compared with PBS or normal mouse IgG (Figures 6A-C). The antibody treatment did not affect the body weight during the experiment (Figure 6D). The expression of TM4SF5 in colon tumor tissue was confirmed by immunostaining with the anti-TM4SF5 antibody (Figure 6E). Analysis of the results of xenograft experiments revealed that the anti-TM4SF5 monoclonal antibody targeting colon tumor cells can decrease tumor growth *in vivo*.

### Anti-TM4SF5 monoclonal antibody inhibits colon cancer growth in an allograft mouse model

**Figure 7:**
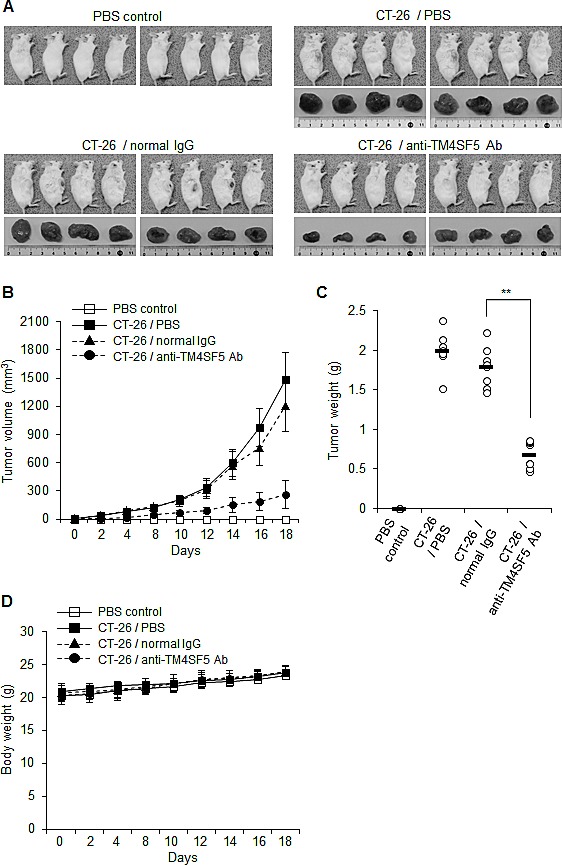
Therapeutic efficacy of the anti-TM4SF5 monoclonal antibody against colon tumor growth in an allograft mouse model A mouse allograft model was established by implantation of CT-26 cells in BALB/c mice. PBS, normal IgG or the anti-TM4SF5 monoclonal antibody was injected intraperitoneally into the tumor bearing mice when the tumors reached 5 mm in diameter, and tumor growth was monitored for 18 days (n=8 each). Control mice injected with PBS are shown as a control (n=8). A. Macroscopic appearance of tumor bearing mice and dissected tumor tissues. B. Tumor volume (width^2^ x length/2). Data are represented as mean + standard deviation. C. Individual tumor weights. Mean values are indicated as a horizontal bar. D. Individual body weights for each treatment group. Data are represented as mean + standard deviation. **p<0.01, mean ± SD.

To investigate the effect of anti-TM4SF5 antibody on colon tumors, we used mouse CT-26 cells and a mouse tumor allograft model. As shown in Figures 7A-C, treatment with the anti-TM4SF5 antibody significantly suppressed the progression of colon tumors derived from CT-26 cells. The antibody treatment did not affect the body weight during the experiment (Figure 7D). Together these experiments suggest that the anti-TM4SF5 monoclonal antibody can inhibit growth of colon tumors in an allograft mouse model.

## DISCUSSION

It has been reported that TM4SF5 mRNA is expressed in colon carcinoma, pancreatic tumors, and HCC [33]. We recently investigated the expression of the TM4SF5 protein in human HCC specimens using the anti-TM4SF5 monoclonal antibody and found that TM4SF5 protein was expressed in all of the 105 HCC tissues examined [42]. In addition to HCC, TM4SF5 expression was also detected in colon cancer and pancreatic cancer tissues [42]. Here, we investigated expression of the TM4SF5 protein in colon cancer tissues in detail and confirmed that the TM4SF5 protein is overexpressed in human colon cancer tissues. In addition, we investigated *in vitro* and *in vivo* effects of anti-TM4SF5 antibody using colon cancer cell lines and mouse models.

This is the first report directly showing expression of TM4SF5 protein in a large number of colon cancer tissues. Based on the immunohistochemistry data of colon cancer tissue microarrays, TM4SF5 expression was detected in almost all of the colon cancer tissue samples we examined, at various expression levels (Table 1). The expression of TM4SF5 in some colon cancer cell lines was also examined. Expression of TM4SF5 is thus likely to be associated with onset or progression of colon cancer cells and TM4SF5 can be a target to treat colon cancer.

We also confirmed that the anti-TM4SF5 antibody inhibited growth of human colon cancer cell lines expressing TM4SF5. Furthermore, we found that the expression of E-cadherin and β-catenin was enhanced by anti-TM4SF5 antibody. These results suggest that treatment with anti-TM4SF5 antibody restores contact inhibition, resulting in reduced cell growth. However, some mechanisms of action induced by anti-TM4SF5 antibody in colon cancer appear to be different from HCC. For example, nuclear localization of p27^kip1^ was detected in HCC cells treated with the anti-TM4SF5 antibody; however a prominent difference after treatment of colon cancer cells with anti-TM4SF5 antibody was not observed. Considering a previous report that p27^kip1^ alterations such as cytoplasmic p27^kip1^ localization or p27^kip1^ loss are associated with superior prognosis of colon cancer patients [44], functional recovery of p27^kip1^ in the nucleus may not be a viable target of anti-tumor activities in colon cancer. Nonetheless, we can conclude that the anti-TM4SF5 antibody may have therapeutic effects on colon cancer.

We therefore validated anti-TM4SF5 monoclonal antibody as an efficacious therapeutic against colon cancer *in vivo* using mouse xenograft and allograft models. To confirm the anti-tumor activity of the anti-TM4SF5 monoclonal antibody against colon cancer *in vivo*, we administered the anti-TM4SF5 antibody into mice bearing tumors pre-established by injection with human or mouse colon cancer cells. The anti-TM4SF5 monoclonal antibody was specifically localized in the tumor tissues (Figure 5) and significantly suppressed tumor growth without prominent side effects (Figures 6 and 7). The obtained results demonstrate that this anti-TM4SF5 monoclonal antibody has therapeutic effects in mouse models of colon cancer, and suggest that injection with anti-TM4SF5 antibody can be an efficacious therapeutics to treat colon cancer in humans.

Considering that TM4SF5 expression was detected also in pancreatic cancer tissues [33,42], we can extend the present research to investigate the expression of TM4SF5 protein in a number of pancreatic cancer tissues and evaluate the efficacy of the anti-TM4SF5 antibody as a therapeutic in a mouse model. Through such research, we can validate the strategy described here to treat pancreatic cancer in the future. As pancreatic cancer is notoriously difficult to treat, the outcome of such research would provide valuable information. Recently, high expression of TM4SF5 in esophageal cancer tissues was reported: a high level of TM4SF5 expression was detected in about 45% of evaluated cases and high TM4SF5 expression was associated with cancer progression and poor patient survival [45]. It is thus possible that anti-TM4SF5 antibody also has anti-cancer effects on esophageal cancer.

To strengthen the anti-cancer effects of the targeted therapy using the anti-TM4SF5 antibody, development of an antibody-drug conjugate may be a better choice, as previously reported [46,47]. For rational and safer antibody therapeutics, tremendous efforts to understand the action mechanism, produce humanized antibodies, and validate *in vivo* effects of the antibodies in detail are warranted.

## MATERIALS AND METHODS

### Production of the mouse anti-human TM4SF5 monoclonal antibody

As described previously [37], we obtained hybridoma cells producing the anti-hTM4SF5 peptide-specific monoclonal antibody after immunization of BALB/c mice with the hTM4SF5R2-3 peptide derived from human TM4SF5 (^138^NRTLWDRCEAPPRV^151^) and CpG-DNA coencapsulated in a DOPE:CHEMS complex. The anti-TM4SF5 monoclonal antibody was purified from the ascitic fluid by protein A column chromatography. As the isotype of anti-TM4SF5 monoclonal antibody is IgG2a, the real control is normal IgG2a. However, we previously confirmed that normal IgG can be used as a control instead of IgG2a [42]. Therefore, we used normal IgG or IgG2a as an antibody control for *in vitro* and *in vivo* experiments.

### Tissue microarrays and immunohistochemistry

For the colon cancer tissue analysis, formalin-fixed, paraffin-embedded AccuMax tissue arrays were purchased from ISUABXIS with the approval of the Institutional Review Board in Hallym University. The colon cancer tissue array (A203VII) is composed of 45 cases of colon cancer tissues in duplicate and 8 normal colon tissues. The slides were deparaffinized with xylene and rehydrated in ethanol, and endogenous peroxidase activity was blocked with 3% hydrogen peroxide for 15 min. For antigen retrieval, all sections were boiled in a citrate buffer (pH 6.0) (ScyTek Laboratories) for 15 min. The slides were incubated overnight in PBST (PBS, Tween 0.2%) containing the anti-TM4SF5 monoclonal antibody (10 μg/ml) at 4°C, followed by incubation with biotinylated anti-mouse IgG antibody (Histostain Plus kit, Invitrogen). They were sequentially reacted with streptavidin-conjugated peroxidase, 3',3'-diaminobenzinidine (0.5 mg/ml) and hydrogen peroxide and then counterstained with hematoxylin. After rinsing, the sections were mounted, dehydrated, and covered with cover slips. All images were examined using a Nikon Eclipse E-200 microscope. The percentages of cells expressing TM4SF5 were calculated as the number of TM4SF5-positive cells divided by the total number of cells in each tumor type.

### Cell culture

The human colon cancer cell lines HT-29, LoVo, and HCT116, and the mouse colon cancer cell line CT-26 were obtained from the Korean Cell Line Bank. CT-26 cells were maintained in a DMEM medium containing 10% fetal bovine serum (FBS; Hyclone), 2 mM glutamine, 100 U/ml penicillin, and 100 μg/ml streptomycin. The other cell lines were maintained in an RPMI 1640 medium with 10% FBS, 25 mM HEPES, 100 U/ml penicillin, and 100 μg/ml streptomycin. Cells were cultured at 37°C in an incubator containing 5% CO_2_.

### Detection of TM4SF5 mRNA expression in cell lines

To analyze the TM4SF5 expression, we performed RT-PCR. Total RNAs were extracted with an RNeasy Mini Kit (Qiagen), and the cDNA was synthesized as described previously [38]. The standard PCR reaction was performed for 25 cycles with the following primer sets: mouse GAPDH, 5'-ATGGTGAAGGTCGGTGTGAACG-3' and 5'-GTTGTCATGGATGATCTTGGCC-3' (501 bp); mouse TM4SF5, 5'-CGCTTACTTGCGAAATGACA-3' and 5'-TTTCCTGCAATCGCCACACA-3' (174 bp), human β-actin, 5'-GGGTCAGAAGGATTCCTATG-3' and 5'-CCTTAATGTCACGCACGATTT-3' (500 bp); human TM4SF5, 5'-AGCTTGCAAGTCTGGCTCAT-3' and 5'-GCTGGATCCCACACAGTACT-3' (408 bp).

### MTT assay

To evaluate the effect of the antibody on the growth of cells, an MTT assay was performed as described previously [48, 49]. Cells were treated with PBS, normal IgG, or anti-TM4SF5 monoclonal antibody (10 μg/ml) for up to 5 days. The MTT (3-(4,5-dimethylthiazole-2-yl)-2,5-diphenyl tetrazolium bromide, Sigma-Aldrich) solution was added to each well and then the plates were incubated for 4 h at 37°C. The medium was removed, and the formazan crystals were solubilized in DMSO. The color development was measured using a spectrophotometer at 595 nm with a reference wavelength of 650 nm.

### Confocal microscopy

Cells were cultured on glass cover slips in 4-well plates 18 h prior to treatment with the anti-hTM4SF5 monoclonal antibody or control IgG (10 μg/ml). After treatment with the antibody for the indicated time periods, the cells were fixed with 4% paraformaldehyde, permeabilized with PBS containing 0.1% Triton X-100, and stained with the anti-E-cadherin antibody (rabbit polyclonal Ab, Santa Cruz Biotechnology, sc-7870) or with anti-β-catenin antibody (rabbit polyclonal Ab, Upstate Biotechnology, #06-734), for 1 h. After extensive washing in PBS containing 0.1% Triton X-100, the samples were incubated with Alexa Flour 488 or Alexa Flour 594-conjugated goat anti-rabbit IgG (Invitrogen) for 1 h. The nuclei were stained with Hoechst 33258, and the mounted samples were scanned with an LSM 710 (Carl Zeiss).

### Western blotting

Proteins were separated by SDS-PAGE and transferred onto PVDF membranes (Pall Corporation). The membranes were incubated with primary antibody for 2 h, washed with PBST, and incubated in HRP-conjugated secondary antibody (Jackson ImmunoResearch Laboratories) for 1 h at room temperature. Blots were developed using ECL reagents (Intron Biotechnology).

### Biodistribution imaging *in vivo*

5 mg/ml of the anti-TM4SF5 monoclonal antibody and IgG2a control (Bethyl Laboratories) in PBS solution was adjusted to contain 50 mM borate buffer (pH 8.5). The antibodies were conjugated with DyLight 755 and purified using a DyLight 755 Antibody Labeling Kit (Thermo Scientific). Fifty micrograms of DyLight 755-labeled anti-TM4SF5 antibody or DyLight 755-labeled IgG2a control were injected into the intraperitoneal cavity of BALB/c control mice or mice bearing CT-26 cell derived tumors. The distribution of the DyLight 755-labeled antibodies was quantified by *in vivo* fluorescence using a real-time IVIS imaging system 200 (Xenogen Corp.). To investigate the localization of the injected DyLight 755-labeled antibodies in colon tumor tissues, the tissues were removed at 72 h. The frozen tissues were cut into 4-μm-thick slices using a cryostat and stained with SYTOX Green dye for nuclei, and the mounted samples were examined with an LSM 710.

### Animals

Mice were maintained under specific pathogen-free conditions in the Experimental Animal Center of Hallym University. Four-week-old male BALB/cAnCrj-nu/nu mice and BALB/c mice were purchased from OrientBio. Our animal studies were performed according to the recommendations in the Guide for the Care and Use of Laboratory Animals of the National Veterinary Research & Quarantine Service of Korea. All procedures involving animal studies were approved by the Institutional Animal Care and Use Committee of Hallym University (Permit Number: Hallym 2011-89, Hallym 2012-81-1). The mice were sacrificed under Zoletil 50+Rompun anesthesia with all efforts to minimize suffering.

### Colon cancer mouse model

For the allograft assays, thirty BALB/c mice were inoculated subcutaneously in the dorsal right flank with 5x10^6^ CT-26 cells in 50% Matrigel as previously described [40]. When tumors reached 5 mm in diameter, the mice were randomly divided into three treatment groups (8 mice/group): PBS, IgG control, and the anti-TM4SF5 monoclonal antibody. The antibodies (25 mg/kg) were injected twice weekly into the intraperitoneal cavity. Tumor diameters were measured using calipers at 2 day intervals for 18 days following the injection of CT-26 cells, and tumor volumes were calculated using the formula width^2^ × length/2. Finally, the mice were sacrificed 18 days after CT-26 cells injection and the tumors were weighed. For the xenograft assays, thirty BALB/cAnCrj-nu/nu mice were inoculated subcutaneously in the dorsal right flank with 5x10^6^ HT-29 cells containing 50% Matrigel. When tumors reached 5 mm in diameter, the mice were randomly divided into three treatment groups (8 mice/group): PBS, IgG control, and the anti-TM4SF5 monoclonal antibody. Six mice injected with neither cancer cells nor antibody were used as a control. The antibodies (25 mg/kg) were injected twice weekly into the intraperitoneal cavity. Tumor diameters were measured using calipers at 5 day intervals for 25 days following the injection of HT-29 cells, and tumor volumes were calculated using the formula width^2^ × length/2. Finally, the mice were sacrificed 30 days after HT-29 cells injection and the tumors were weighed.

### Histology and immunohistochemistry

The tumors were removed and fixed in a 4% buffered formalin solution overnight, embedded in paraffin using standard methods, and cut into 5-μm-thick sections. The sections were deparaffinized and then stained with hematoxylin and eosin (H&E). To evaluate the expression of TM4SF5, the deparaffinized sections were stained with the anti-TM4SF5 monoclonal antibody (10 μg/ml), according to standard procedures using a Histostain Plus kit. The samples were then counterstained with hematoxylin. All images were examined using a Nikon Eclipse E-200 microscope.

### Statistical analysis

Results are expressed as mean + standard deviation. Statistical significance between two samples was evaluated using the Student's *t* test. A *p*-value of <0.05 was taken as statistically significant.
